# Identification of *Escherichia coli* and Related Enterobacteriaceae and Examination of Their Phenotypic Antimicrobial Resistance Patterns: A Pilot Study at A Wildlife–Livestock Interface in Lusaka, Zambia

**DOI:** 10.3390/antibiotics10030238

**Published:** 2021-02-26

**Authors:** Emmanuel Kabali, Girja Shanker Pandey, Musso Munyeme, Penjaninge Kapila, Andrew Nalishuwa Mukubesa, Joseph Ndebe, John Bwalya Muma, Charles Mubita, Walter Muleya, Elizabeth Muligisa Muonga, Shuya Mitoma, Bernard Mudenda Hang’ombe, Anuwat Wiratsudakul, Mai Thi Ngan, Eslam Elhanafy, Hala El Daous, Nguyen Thi Huyen, Wataru Yamazaki, Tamaki Okabayashi, Maiku Abe, Junzo Norimine, Satoshi Sekiguchi

**Affiliations:** 1 Quality Assurance Unit, Director General’s Office, Zambia Medicines Regulatory Authority, Lusaka 10101, Zambia; cutrite50@yahoo.co.uk; 2 Department of Disease Control, School of Veterinary Medicine, University of Zambia, Lusaka 10101, Zambia; pandeygs@gmail.com (G.S.P.); mussomunyeme@gmail.com (M.M.); penjanikapila@yahoo.com (P.K.); mukubesaandrew@gmail.com (A.N.M.); j.ndebe@yahoo.com (J.N.); jmuma@unza.zm (J.B.M.); cmubita55@yahoo.co.uk (C.M.); elizabethmuligisa@gmail.com (E.M.M.); 3 Department of Biomedical Sciences, School of Veterinary Medicine, University of Zambia, Lusaka 10101, Zambia; muleyawalter@gmail.com; 4 Department of Environmental Health, School of Health Sciences, Eden University, Lusaka 10101, Zambia; 5 Graduate School of Medicine and Veterinary Medicine, University of Miyazaki, Miyazaki 889-1692, Japan; Matomi1510@gmail.com (S.M.); ngan16a@gmail.com (M.T.N.); hala.ali@fvtm.bu.edu.eg (H.E.D.); 6 Department of Para-Clinical Studies, School of Veterinary Medicine, University of Zambia, Lusaka 10101, Zambia; Mudenda68@yahoo.com; 7 Department of Clinical Sciences and Public Health, Faculty of Veterinary Science, Mahidol University, Nakhon Pathom 73170, Thailand; anuwat.wir@mahidol.edu; 8 Faculty of Veterinary Medicine, Vietnam National University of Agriculture, Hanoi 100000, Vietnam; 9 Faculty of Veterinary Medicine, Benha University, Moshtohor, Toukh, Qalyubia 13736, Egypt; dr.eslam.elshehry@gmail.com; 10 National Institute of Veterinary Research, Hanoi 100000, Vietnam; nguyenhuyen150187@gmail.com; 11 Center for Southeast Asian Studies, Kyoto University, Kyoto 606-8501, Japan; yamazaki@cseas.kyoto-u.ac.jp; 12 Department of Veterinary Science, Faculty of Agriculture, University of Miyazaki, Miyazaki 889-2192, Japan; okbys81@cc.miyazaki-u.ac.jp (T.O.); nori@cc.miyazaki-u.ac.jp (J.N.); 13 Centre for Animal Disease Control, University of Miyazaki, Miyazaki 889-2192, Japan; 14 Education and Research Center for Mathematical and Data Science, Hokkaido University, Hokkaido 060-0812, Japan; mike_abe@cris.hokudai.ac.jp

**Keywords:** antimicrobial resistance, domestic animals, *Escherichia coli*, molecular detection, public health, wildlife, wildlife–livestock–human interface, Zambia

## Abstract

A cross-sectional study was used to identify and assess prevalence and phenotypic antimicrobial resistance (AMR) profiles of *Escherichia coli* and other enterobacteria isolated from healthy wildlife and livestock cohabiting at a 10,000 acres game ranch near Lusaka, Zambia. Purposive sampling was used to select wildlife and livestock based on similarities in behavior, grazing habits and close interactions with humans. Isolates (*n* = 66) from fecal samples collected between April and August 2018 (*n* = 84) were examined following modified protocols for bacteria isolation, biochemical identification, molecular detection, phylogenetic analysis, and antimicrobial susceptibility testing by disc diffusion method. Data were analyzed using R software, Genetyx ver.12 and Mega 6. Using Applied Profile Index 20E kit for biochemical identification, polymerase chain reaction assay and sequencing, sixty-six isolates were identified to species level, of which *Escherichia coli* (72.7%, 48/66), *E. fergusonii* (1.5%, 1/66), *Shigella sonnei* (22.7%, 14/66), *Sh. flexinerri* (1.5%, 1/66) and *Enterobacteriaceae bacterium* (1.5%, 1/66), and their relationships were illustrated in a phylogenetic tree. Phenotypic antimicrobial resistance or intermediate sensitivity expression to at least one antimicrobial agent was detected in 89.6% of the *E. coli*, and 73.3% of the *Shigella* isolates. The *E. coli* isolates exhibited the highest resistance rates to ampicillin (27%), ceftazidime (14.3%), cefotaxime (9.5%), and kanamycin (9.5%). Multidrug resistance (MDR) was detected in 18.8% of *E. coli* isolates while only 13.3% *Shigella* isolates showed MDR. The MDR was detected among isolates from impala and ostrich (wild animals in which no antimicrobial treatment was used), and in isolates from cattle, pigs, and goats (domesticated animals). This study indicates the possible transmission of drug-resistant microorganisms between animals cohabiting at the wildlife–livestock interface. It emphasizes the need for further investigation of the role of wildlife in the development and transmission of AMR, which is an issue of global concern.

## 1. Introduction

Antimicrobial resistance (AMR) occurs when bacteria, viruses, fungi and parasites change over time and no longer respond to antimicrobial medicines (including antibiotics, antivirals, antifungals and antiparasitics) making infections harder to treat and increasing the risk of disease spread, severe illness and death [1]. According to WHO, AMR is occurring everywhere in the world, compromising humans’ ability to treat infectious diseases, and undermining many other advances in health and medicine [2]. Globally, it is estimated that antimicrobial resistance (AMR) will be responsible for 10 million deaths per year by 2050 if steps are not taken immediately to combat and prevent the spread of AMR. In addition, the cumulative economic cost of AMR, estimated to be 100 trillion USD, is more than one and a half times the annual world gross domestic product as of 2015 [2,3]. 

Therefore, the emerging threat of AMR to public health continues to attract significant attention worldwide [2,3,4]. In response to this threat, a global initiative led by the World Health Organization (WHO), the Food and Agricultural Organization and the World Organisation for Animal Health (OIE), encompassing a global action plan, has been implemented to combat AMR [2]. Various factors contribute to the emergence of AMR. These include: limited knowledge of the risk of AMR, inappropriate use of antimicrobial agents in humans, animals and plants, including overprescribing and dispensing [3,5], poor drug quality and lack of drug quality control and monitoring/testing facilities [3,6], lack of adherence to good distribution practices and inadequate regulation of distribution channels/facilities [6,7], patients not completing the entire prescribed treatment course, and poor/inadequate healthcare facilities for provision of clinical services to humans, animals and plants [3].

Specifically, limited knowledge of the risk of AMR at various levels within global and local ecosystems will continue to hamper effective action for the control of AMR. For instance, recent studies on AMR have shown that commensal microorganisms belonging to families such as Enterobacteriaceae are becoming increasingly important in the transmission of resistance genes [1,5,8]. Indicator organisms such as *Escherichia coli* are receiving increased research attention based on observation that most commensal microorganisms display inherent resistance to specific antimicrobial agents [1,8]. The importance of these organisms to AMR risks is reinforced by recent findings showing that the use of broad-spectrum antibiotic treatments tends to select for resistant microorganisms within the human microbiome [9,10]. With the ability to transmit AMR genes horizontally via mobile genetic elements, commensal bacteria may play a significant role in the development and spread of AMR among other microorganisms [5,11]. This becomes a concern when transmission occurs from commensal to pathogenic species, and/or when commensal microorganisms acquire virulence determinants through mutations, making them pathogenic. The AMR transmission dynamics become even more significant when it implicates immunocompromised individuals, due to chronic and lifelong conditions such as HIV/AIDS, diabetes and cancer, requiring them to depend on long-term treatments [1].

Relatively little is known about the prevalence and development of AMR in bacteria from wild animals or the environment, although there is an increasing number of reports on multidrug-resistant (MDR) organisms in natural environments [12]. An understanding of the occurrence of AMR in environmental pathogens and commensals is important, especially when there are close interactions among wildlife, livestock and humans. This is even more relevant given our increasing knowledge of horizontal transmission of resistance genes via mobile elements among microorganisms [5,11], and the likelihood of preservation of microorganisms in wild animals and the environment [13]. The increasing interactions among humans, livestock and wildlife increase the public health significance of microorganisms that can be transmitted from wildlife and the environment to humans and livestock [13,14,15,16,17,18,19]. Therefore, the purpose of the current study was to investigate the prevalence and types of AMR amongst bacteria from apparently healthy wildlife and livestock cohabiting at a game ranch in Lusaka, Zambia.

## 2. Results

### 2.1. Microbiological, Biochemical and Molecular Analysis for Identification of Enterobacteriaceae Fecal Isolates

A total of 84 fecal samples were collected, from which 66 presumptive *E. coli* isolates were obtained. From API biochemical tests of the 66 presumptive *E. coli* isolates, 59.1% (*n* = 39) were identified as *E. coli* Group 1, one isolate was identified as *Burkholderia cepacia* and one isolate was identified as *Kluyvera* species. The remaining 25 isolates (37.9%) could not be conclusively identified using available API 20E testing kits (Table 1). 

Using PCR and cycle sequencing, the majority of the isolates were identified to strain level. Among isolates from impala, *E. coli* strains detected included *E. coli* FR–2, *E. coli* LD93–1, *E. coli* ECPF–16, and *E. coli* WP2–S18–ESBL–07. Among isolates from cattle, *E. coli* strains included *E. coli O157* CFSAN 076619, *E. coli* 824422, and *E. coli* LD93–1. In ostrich, *E. coli* RHB04–C05 and *E. coli* YJ4 strains were detected, while in goats, *E. coli* UF–153 and *E. coli* WP5–W18–ESBL 11, and in pigs, *E. coli* PB7 DCRUST SK strains were detected. *Shigella sonnei* SE6–1 strain was detected at least in one of the samples from each animal species, while *Shigella* spp. NCCP–460 was detected in a sample from pig. *Shigella flexinerri* SD5 strain was detected in a sample from cattle. 

### 2.2. Phylogenetic Analysis

**Figure 1:**
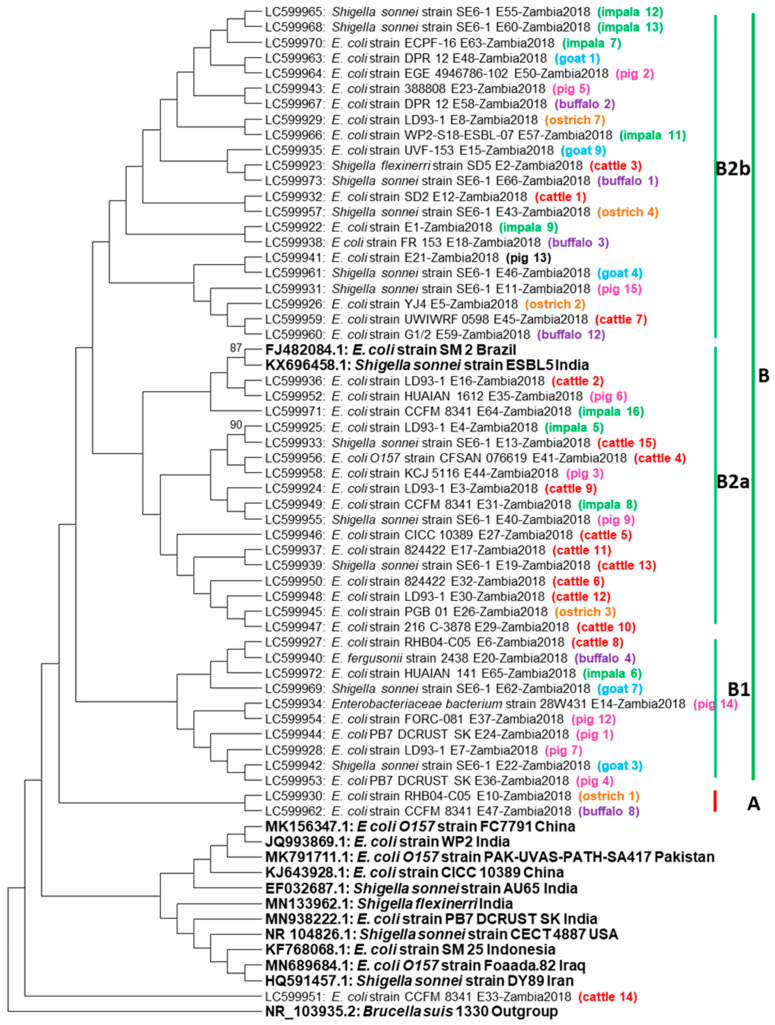
Phylogenetic tree. The 16S rRNA gene neighbor joining phylogenetic tree based on 500 bp nucleotide sequences from *Escherichia coli* and *Shigella* isolated from wildlife and domestic animals. The tree was constructed using mega 6 using 1000 bootstrap replicates as confidence interval. The color codes represent origin from the same animal species. The isolates under study were separated into two clusters namely A and B, with cluster A having only 2 isolates from wild animals (buffalo and ostrich) while cluster B was divided into minor clusters B1 and B2 with B2 further dividing into B2a and B2b and most isolates were in this cluster.

Phylogenetic analysis of the16S rRNA gene sequences showed that all the isolates under study separated into two clusters namely A and B (Figure 1). 

Cluster B was divided into minor clusters B1 and B2 with B2 further dividing into B2a and B2b. Within cluster A, only two wildlife sequences were present, these being *E. coli* originating from a buffalo and an ostrich. On the other hand, cluster B consisted of sequences from both wildlife and domestic animals as well as *E. coli* and *Shigella* reference sequences from Brazil and India. Most sequences in cluster B were *E. coli* with a few being *Shigella*. Many sequences in cluster B1 were *E. coli* obtained from pigs (*n* = 4) (Figure 1). Within cluster B2a, most sequences originated from cattle and these were two *Shigella* and eight *E. coli* sequences, respectively, while in cluster B2b, most sequences originated from impala (*n* = 5) with two being *Shigella* and the remaining three being *E. coli.* Overall, in cluster B, clustering based on type of organism or origin of the sample was not observed, instead sequences from different animals and bacterial organisms formed similar clusters, indicating the close similarity of the 16S rRNA gene in *E. coli* and *Shigella* from both wildlife and domestic animals (Figure 1).

### 2.3. Antimicrobial Susceptibility Testing

Antimicrobial susceptibility testing (AST) results indicated that *Shigella* isolates showed resistance or intermediate sensitivity to seven of the thirteen antimicrobials screened in this study, while the *E. coli* isolates showed resistance or intermediate sensitivity to eleven of the thirteen antimicrobials. Intermediate resistance to at least one antimicrobial agent was shown by 89.6% (*n* = 43) of the *E. coli* isolates and 73.3% (*n* = 11) of the *Shigella* isolates. Isolates obtained from impala showed the greatest diversity in antibiotic resistance, exhibiting resistance to eight different antimicrobial agents in total. In comparison, isolates from buffalo showed the least diversity, exhibiting resistance to none of the antimicrobial agents tested (Table 2). Of the *E. coli* isolates, 18.8% (*n* = 9) were MDR, while 1.3% (*n* = 2) of the *Shigella* isolates were MDR. The higher number of MDR isolates were recovered from impala (*n* = 3), while others were isolated from cattle (*n* = 2), ostrich (*n* = 2), pig (*n* = 2) and goat (*n* = 2) samples. *Shigella* MDR isolates were each recorded in ostrich and pig (Table 3). The MDR was observed in various combinations, including penicillins, cephalosporins, and quinolones combination (most commonly detected); penicillins, cephalosporins, and aminoglycosides combination; as well as penicillins, cephalosporins, and carbapenems combination. 

## 3. Discussion

In this study, several bacteria strains were detected in wildlife using both biochemical and molecular techniques. A few similar studies have previously been conducted to examine AMR in apparently healthy pastoralist cattle in an interface area in Zambia by Mubita et al. [20], and in a more recent study, Mubita et al. [21] also applied a combined approach similar to this study (molecular characterization and phenotypic detection of AMR) in examining AMR in *Salmonella* isolates at the wildlife–livestock interface in Zambia. This combined approach has, however, not been applied in examining AMR in *E. coli* isolates at the interface in Zambia.

In Impala, for instance, there has not been any reported cases in which *E. coli* FR–2, *E. coli* LD93–1, *E. coli* ECPF–16, and *E. coli* WP2–S18–ESBL–07 strains were detected. These *E. coli* strains may not necessarily be commensal, even though they were isolated from apparently healthy animals. Specifically, the isolation of extended beta-lactamase producing bacteria in animals in which antimicrobials may not have been used might be evidence relevant in elucidating the role of wildlife in the transmission and maintenance of antimicrobial resistance genes in the wild. The isolation of *Shigella sonnei* SE6–1 strain in all animal species sampled, domesticated and wild alike, is also a first report in Zambia at a wildlife–livestock interface. This is an important finding as *Shigella* species have been reported to be among the common pathogens found in animals and are responsible for causing diarrheal diseases in humans in some African countries [22]. 

The use of the combined approach made it possible to identify isolates to strain level. Difference in identity of some isolates at species level were noted, between phenotypic and molecular identification. It was interesting to note that on selective media, all the 66 isolates showed phenotypes characteristic of *E. coli*. In most resource-limited settings, these isolates would be presumed to be *E. coli*, and this may result in suboptimal handling of the otherwise more potent and pathogenic organisms. Using the API system, 37.9 % of the isolates could not be categorically identified. This was either because the organisms identified were not in the API data base, or the identity confidence was less than 90 %. The cytochrome oxidase test, and supplementary tests (reduction of nitrates to nitrites (NO_2_) and N_2_ gas (N_2_); motility (MOB); oxidation of glucose (OF-O); fermentation of glucose (OF-F)) were not conducted, as confirmatory identification was done using available PCR, and sequencing techniques. Two isolates identified by API system as *Burkholderia cepacia* and *Kluyvera* species, both were identified as strains of *E. coli* on genotypic testing. In the final instance, the genotypic identities were relied upon. 

In addition, the clustering on phylogenetic analysis (Figure 4) implied that the examined sections of the 16S rRNA gene in both the *E. coli* and *Shigella* bacteria were very similar and illustrates how closely related *E. coli* and some *Shigella* species are, based on the highly conserved 16S rRNA gene. From a One Health point of view, the close relation between *E. coli* and certain *Shigella* species has been illustrated for some time. For instance, in 2000, Johnson [23] describes the role of *Shigella flexinerri* and *Shigella sonnei* in causing endemic shigellosis in developed countries, while *Shigella dysenteriae* was reported to be responsible for the same disease in developing countries. Our findings, however, show that *Shigella sonnei* and *Shigella flexinerri* isolated from apparently healthy animals have the potential to cause shigellosis in humans, especially considering that the common routes of transmission of these obligate pathogens are the fecal–oral route, with food, water, fomites, insects, and direct contact [23], all of which are possible at the wildlife–livestock–human interface. Further, the presence of bacteria that are so closely related, and exhibiting AMR may complicate accurate diagnosis and institution of effective treatment, especially in settings where both diagnostic and treatment options are limited. The detection of MDR in some of these isolates greatly increases the importance of their presence in apparently healthy animals, from the above mentioned One Health viewpoint. 

In this study, evidence of AMR occurring at the wildlife–livestock interface was demonstrated. Of note were the observed higher levels of AMR amongst isolates from impala compared with domestic animals. These preliminary findings are uniquely important given that the wildlife–livestock–human interface is a fertile location for the exchange of disease-causing microorganisms, including pathogens of animal health and zoonotic importance [14,15,16,17,18,19,24,25]. Zoonotic diseases of global importance have previously been shown to be transmitted at this interface, including rabies, Ebola, anthrax, trypanosomiasis and tuberculosis [14,15,16,17,18,19,26,27,28,29]. This complex disease situation is compounded by the presence of AMR [1,8]. With the implication of transmission via commensal microorganisms such as *E. coli*, AMR genes in these species become a grave concern. In our view, the transmission of resistance at the wildlife–livestock interface complicates the fight against AMR in human pathogens, as it has done for the treatment of many important animal and zoonotic diseases, because wild animals are a stable reservoir of microbial pathogens. This is further reinforced by the fact that AMR is inherent in every treatable disease complex. 

AMR amongst bacteria of wildlife origin has been detected in several countries but has not been widely reported in Zambia [21,30]. In our study, higher levels of resistance were observed amongst isolates from impala and ostrich. This is an interesting finding because antimicrobials are rarely used in wild animals. It was, therefore, hypothesized that resistance may have been acquired via either direct contact with livestock and humans or indirectly via the environment, as levels of resistance were higher in isolates from both wild (impala and ostrich) and domestic (cattle and goat) animals. Drinking water from dams and streams, which drain from communities around the game ranch, is another possible route for the acquisition of resistance. In this case, it would have been expected that the prevalence of AMR amongst isolates from buffalo samples would have been higher. In addition, our findings were contrary to our expectation that the prevalence and patterns of AMR in isolates from buffalos would be alike those observed in cattle as they have similar grazing patterns. It was thought that the solitary and aggressive nature of wild buffalos, which minimizes close contact with other wildlife, domestic animals and humas, may have been a factor in the lower levels of resistance detected in the current study. Despite this, the detection of resistance at the levels observed in wildlife-derived isolates in the current study is a cause for concern. Most microorganisms found in the environment and in wild animals are highly resilient and can survive harsh environmental conditions, allowing them to persist in the environment. Their preservation in the community can also be influenced by wildlife conservation policies and practices [13]. 

The prevalence of AMR among isolates from pigs was relatively low. However, the pigs are raised under an intensive production system, which lowers the risk of transmission of AMR microorganisms from other animals. In this case, resistance would likely be acquired through contact with human attendants and through inappropriate antimicrobial use. The low levels of resistance in isolates from goats were interesting and contrary to our expectations. At the study site, goats were used for tick control and had more frequent contact with other livestock, wildlife and humans, which would make them more likely to acquire AMR microorganisms.

Our findings raise concerns about some wildlife products. In most instances, strict food safety procedures are not followed in the preparation and trade of game meat [13]. For instance, poachers of wild animals for game meat and trophies often do not follow approved meat processing procedures [13]. Therefore, subsequent handlers and eventual consumers of the illicit products risk infection with the resistant microorganisms. This problem can be compounded in situations where food control systems are poor, and hygiene in food production and processing is compromised, with bacterial contamination occurring at high rates [31]. Trophy hunters and handlers may also have some risk of contracting these resistant bacteria through direct contact. Observance and enforcement of basic biosafety, biosecurity, and food hygiene and safety measures in handling products of wild animal origin could address most of these risks.

Our study showed that the highest levels of resistance were expressed against ampicillin in most *E. coli* isolates. Resistance to third generation cephalosporins was higher amongst *E. coli* isolates obtained from impala, cattle and pig samples, with the greatest number of resistant isolates originating from impala. This finding was deemed important, as the tested third generation cephalosporins were all relatively new members with enhanced activity against Gram-negative bacteria, including β-lactamase-producing strains [32,33]. This is concerning given the higher proportion of *E. coli* isolates, some of which may be pathogenic and could also contribute to the transmission of resistance to other bacteria. There were a number of isolates which showed resistance to penicillin and also to some cephalosporins, hence considered as extended-spectrum β-lactamase-producing, which is particularly important given that β-lactam antimicrobials are classified by the WHO as being amongst the highest priority of the critically important antimicrobials for human medicine, and by the OIE as critically important veterinary antimicrobial agents [34,35]. In most recent studies conducted in Zambia, resistance to these antimicrobials was common, with similar trends observed worldwide [29,36]. 

The prevalence of MDR was slightly higher among *E. coli* isolates (18.8 %) compared with the *Shigella* isolates (13.4%). The high levels of MDR among these isolates justify the need to further investigate the mode(s) of transmission of the corresponding resistance gene(s) and identify the elements responsible for the resistance at the molecular level. However, in the absence of requisite capacities to conduct molecular characterization, the results obtained in this study could be useful for estimating the prevalence of AMR in interface areas. Specifically, our results showed that although some isolates were resistant to up to six individual antimicrobials, none of the isolates were resistant to more than four classes of antimicrobials, an important indicator when considering availability of appropriate antimicrobial treatment options for use in both humans and animals. It is important, however, to note that the observed MDR profiles could rapidly change considering the variety of drug resistance phenotypes observed in this study and the ease of horizontal transfer of resistance determinants among bacteria [5,8,11].

## 4. Materials and Methods

### 4.1. Ethical Consideration

This study was designed as part of a larger study, for which ethical approval was sought and obtained from the ERES Converge Institutional Review Board in Lusaka, Zambia (Ref: no. 2018–Apr–006). Consent to conduct the study and collect samples from selected wild animals was also obtained from the game ranch management. The sex of the animals was not considered as a specific factor in the current study. The study was conducted according to relevant guidelines for scientific research using animal subjects and was strictly observational.

### 4.2. Study Design

**Figure 2:**
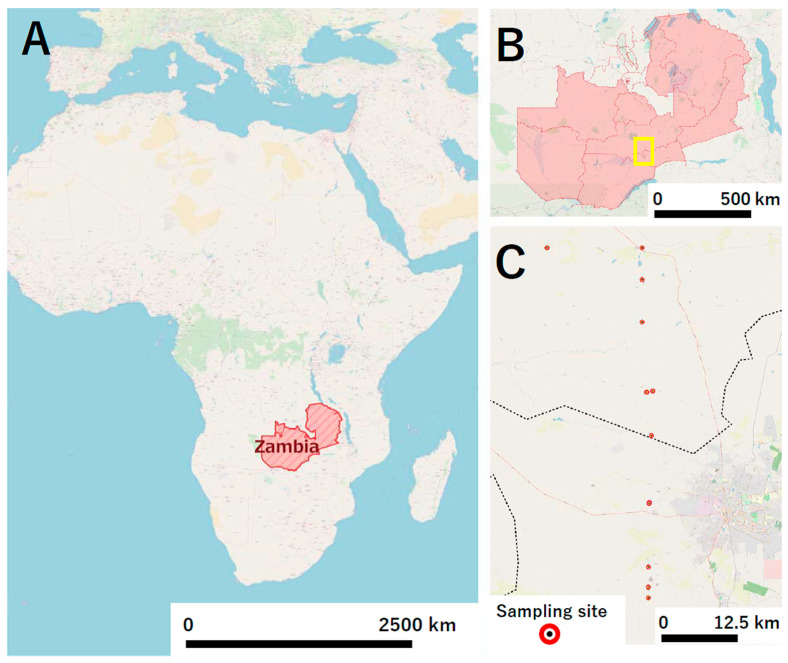
**The location of the study site.** Zambia is a landlocked country located in the Central Southern Africa (**A**); The study site is a game ranch located about 40 km north of Lusaka, Zambia’s capital city (**B**); Fresh fecal samples were collected from sites within 10 km radius of the main office at the game ranch and GPS coordinates were recorded immediately after sample collection (**C**).

A cross-sectional study was used to examine the AMR profiles of bacteria from selected wild animals and livestock at a game ranch located about 40 km north of Lusaka, Zambia’s capital city (Figure 2A,B). The game ranch is completely fenced off, covering an area of about 10,000 acres, and is a conservancy with multiple species of wildlife and livestock, with more than 72 wild animal species reported. In the current study, focus was mainly on antelope and bovine species for sampling, with candidate species including blue wildebeest (*Connochaetes taurinus*), buffalo (*Syncerus caffer*), bushbuck (*Tragelaphus sylvaticus*), eland (*Taurotragus oryx*), hartebeest (*Alcelaphus buselaphus*), impala (*Aepyceros melampus*), Kafue lechwe (*Kobus leche kafuensis*), puku (*Kobus vardonii*), roan antelope (*Hippotragus equinus*), sable antelope (*Hippotragus niger*), and the common Tsessebe (*Damaliscus lunatus*).

Probability assessment, based on population size and frequency of citing at selected locations, was used to determine which wildlife species were most likely to come into close contact with livestock and humans. Grazing patterns were also considered in sample selection, and seasonality was a factor for convenience of sample collection. Impala and buffalo, with estimated park populations of 1000 and 70, respectively, were selected for sampling because they were determined to have been more interactive, and/or showing similar grazing patterns to the livestock kept on the ranch (goats and cattle). Ostriches (*Struthio camelus*) were also selected as they had close contact with the four other selected species, while pigs were selected because they had close contact with humans but were reared in enclosed pig houses. 

### 4.3. Sample Collection

Fresh fecal samples were collected from the target species using the identification method described by Chame [37], between April and August 2018. The research team followed a buffalo herd and identified feces that had been dropped within the previous 4 h. Five herds of impala were also followed, with fecal samples collected upon observation of defecation when the animals stopped. Samples were also collected from a flock of ostriches, and samples were routinely collected from livestock (cattle, goats and pigs). All samples were collected within a 10 km radius of the main office at the game ranch, located at latitude −15.155472 and longitude 28.498028 (Figure 2C). Samples were collected aseptically and placed in sterile sampling bottles before being assigned identification numbers and labelled. Samples were placed in ice cooled boxes and transported for processing at the Microbiology and Public Health laboratories, Department of Disease Control, School of Veterinary Medicine, University of Zambia. For all samples, the process of bacterial isolation and identification was initiated on the day of sample collection (Supplement A). Fecal samples were then stored at −20 °C for further use or reference.

### 4.4. Bacteria Isolation

Approximately 1 g of each fecal sample was suspended in 9 mL of buffered peptone water (Oxoid, Cheshire, England) and incubated at 37 °C overnight (18–24 h) for pre-enrichment. Selection for *E. coli* was conducted by inoculating 10 µL of the enriched suspension onto lactose MacConkey agar (Oxoid) plates using sterile wire loops and incubated at 37 °C overnight. Four presumptive *E. coli* isolates from lactose MacConkey agar were stained using the Gram staining method and further inoculated onto eosin-methylene blue (EMB) agar (Oxoid) and incubating aerobically at 37 °C for 24 h. Up to two presumptive *E. coli* colonies (demonstrating the characteristic metallic green sheen on EMB agar) from each sample were then inoculated onto non-selective blood agar (Oxoid). Atypical *E. coli* colony from each sample was also inoculated onto blood agar and incubated at 37 °C overnight. Isolates were subcultured on nutrient agar to ensure purity. 

### 4.5. Biochemical Identification

Isolates were subjected to biochemical tests by using the API^®^ 20 E Gram-negative Microbial Identification Kit (bioMérieux, Midrand, South Africa) for identification of Enterobacteriaceae to species level. *E. coli* strain ATCC 25922 was used as positive control. Isolates that were not conclusively identified to genus or species level were collectively referred to as “unidentified isolates”.

### 4.6. Molecular Identification

#### 4.6.1. DNA Extraction and Polymerase Chain Reaction

Chromosomal DNA was extracted from purified colonies of every isolate, after it was cultured on nutrient agar. DNA extraction was done using the QIAamp DNA Mini Kit (Qiagen, South Africa). Identification was done using a combination of three primers, 16E1 (forward primer), 16E2 and 16E3 (both reverse primers), targeted at the 16S gene and designed to detect all pathogenic and non-pathogenic *E. coli* strains, *Shigella sonnei* and *Shigella flexineri*, applying a modified PCR procedure as described by Tsen et al. [38]. Each 20 µL PCR mixture contained 1 x PCR buffer (2.0 µL), 260 µM/L dNTP (1.6 µL), 1 µM/l of each primer, 0.5 U of Taq DNA polymerase enzyme (0.1 µL), milli q water (11.9 µL), and template DNA (from sample) (2.0 µL). For each PCR cycle, denaturation, annealing, and extension were carried out at 95.0 °C for 2 min, 60.0 °C for 30 s, and 72 °C for 2 min, respectively. Final extension was done at 72 °C for 10 min. A total of 35 PCR cycles were performed using the Veriti™ 96-Well Thermal Cycler (Applied Biosystems™, Thermo Fisher Scientific, Waltham, MA, USA). PCR products were examined for a fragment of about 584 bp visualized in ethidium bromide-stained 1.5% agarose gel under UV light. Identification of the product band was done by molecular weight maker of the Takara 100 bp DNA Ladder (Takara, Japan).

For isolates that could not be detected using the 16E1/16E2/16E3 primers, the more general primers (P3mod and P5) for identification of bacteria by targeting the 16S gene were used to identify the isolates, using a modified PCR procedure described by Tsen et al. [38]. Each 20 µL PCR mixture contained 1 x PCR buffer (2.0 µL), 260 µM/L dNTP (1.6 µL), 1 µM/L of each primer, 0.5 U of Taq DNA polymerase enzyme (0.1 µL), milli q water (12.3 µL), and template DNA (from sample) (2.0 µL). Each PCR cycle, denaturation, annealing, and extension were carried out at 95.0 °C for 1 min 30 s, 57.0 °C for 1 min 30 s, and 72 °C for 3 min, respectively. Final extension was done at 72 °C for 10 min. A total of 35 PCR cycles were performed using the Veriti™ 96-Well Thermal Cycler from Applied Biosystems™ (Thermo Fisher Scientific, Waltham, MA, USA). A 700 bp fragment was visualized in ethidium bromide-stained 1.5% agarose gel. 

#### 4.6.2. Cycle Sequencing

The PCR product obtained was purified using the DNA purification kit (Promega, USA). The purified PCR products were sequenced using Big dye terminator version 3.1 (Thermo Fischer) according to the manufacturer’s specifications. The sequence PCR products obtained were further purified using the ethanol purification method followed by the denaturation of the purified products and capillary electrophoresis using the ABI 3500 Genetic Analyzer (Applied Biosystems). 

#### 4.6.3. Sequence Analysis

For sequence analysis, raw sequences were initially subjected to blast analysis on the NCBI website (http://blast.ncbi.nlm.nih.gov/Blast.cgi access on 16 January 2021) followed by sequence assembly using the ATGC plug-in incorporated in Genetyx ver. 12. (Genetyx Co., Tokyo, Japan). Previous published representative sequences of *E. coli*, *Shigella sonnei*, *Shigella flexinerri*, and *Brucella* were downloaded from the GenBank and together with the obtained sequences under study, were used to generate a multiple sequence alignment file using Clustal W1.6. The multiple sequence alignment file was further converted to a MEGA file format and utilized to generate a neighbor joining tree with 1000 bootstrap replicates as a confidence interval using MEGA ver. 6 [39]. All the sequences generated in this study have been deposited in the DNA Data Base of Japan with serial numbers LC599922 to LC599973. 

### 4.7. Antimicrobial Susceptibility Testing (AST)

Antimicrobial susceptibility testing was conducted following standard operating procedure developed through the WHO Advisory Group on Integrated Surveillance of Antimicrobial Resistance (AGISAR) project in Zambia. The Clinical and Laboratory Standards Institute (CLSI) 2018 M100–S28 guidelines [40] were utilized for both testing method and interpretation of the AST. For each of the identified isolates and control strain *E. coli* 25922, 1–2 colonies were picked from the blood agar plates, inoculated onto nutrient agar (Oxoid) and incubated at 37 °C for 18 h. Following incubation, 1–2 colonies were picked from each of the nutrient agar plates and transferred into vials containing 4 mL of sterile normal saline (bioMérieux, Midrand, South Africa) to a McFarland turbidity of ~0.5. Each bacterial suspension was then inoculated onto two 100 mm Muller Hinton (Oxoid) agar plates (poured to a depth of 4 mm) to form a lawn. 

Antimicrobial susceptibility test discs (BD BBL Sensi–Disc; BD Biosciences, Franklin Lakes, NJ, USA) were applied to the surfaces of the Muller Hinton plates in two groupings, as follows: Panel 1—ampicillin, 30 µg; ceftazidime, 30 µg; cefotaxime, 30 µg; kanamycin, 30 µg; imipenem, 10 µg; ceftriaxone, 30 µg; amoxicillin/clavulanic acid, 30 µg; Panel 2—nalidixic acid, 30 µg; amikacin, 30 µg; ciprofloxacin, 5 µg; trimethoprim/sulfamethoxazole, 25 µg; gentamicin, 10 µg; chloramphenicol, 30 µg. The antimicrobials were selected based on their availability on the Zambian market and/or their importance to public health globally. 

Plates were then incubated at 37 °C for 18 h. Assessment of antimicrobial susceptibility was carried out manually using digital Vernier calipers by measuring the diameter (mm) of the zone of inhibition against a dark surface. AST results for control strain *E. coli* ATCC 25922 were compared against expected results to ensure the quality of the test. AST results were entered into an excel spreadsheet for interpretation using the Clinical and Laboratory Standards Institute 2018 guidelines [40].

### 4.8. Statistical Analysis

Statistical analysis of generated data was conducted for 2 parts, first for cycle sequence results, and second for antimicrobial susceptibility tests. Descriptive statistics were computed for all variables, while univariable analysis by cross-tabulation using Pearson’s chi-square test was carried out to test for variability among the isolates across animal species [41]. Further analysis of the variability in AMR profiles among isolates across animal species was also conducted. Isolates were considered MDR if they showed resistance to three or more classes of antimicrobials [42]. All data analyses were conducted using R software (version 3.6.0; R Foundation for Statistical Computing, Vienna, Austria). 

### 4.9. Study Limitations

It is acknowledged that this pilot study had several limitations. First, the number of samples investigated was small; larger studies encompassing multiple wildlife–livestock–human interface areas and larger numbers of samples and isolates are recommended to further examine and confirm our findings. Second, while interesting AMR profiles were observed amongst the isolates, further molecular investigation should be conducted to characterize the presence and types of AMR genes responsible for the resistance expressed phenotypically. This information is essential for confirming the mode of resistance and similarities among resistance genes in isolates from wildlife and livestock. Third, the sample size was limited to a single wildlife–livestock interface, and therefore cannot generalize the findings to all interface areas in Zambia. However, these findings are useful for informing further surveillance activities in these areas that are not well studied in terms of AMR. 

## 5. Conclusions

The role of the environment and wildlife in the maintenance and transmission of AMR is of growing concern. By conducting a cross-sectional survey of AMR amongst *E. coli* and closely related enterobacteria isolated from selected wild animals and livestock at a game ranch in Zambia, it was possible to identify the presence and estimate the prevalence of AMR within the sampled population. Our findings indicate that there is indeed a likelihood of transmission of AMR between livestock and wildlife cohabiting closely, although the transmission dynamics could not be explained based on these results. Our study also confirmed the urgent need to further investigate the role of wildlife and the shared environments in the transmission and maintenance of AMR, which is a One Health issue of global concern. This information is important for improving wildlife and livestock management, and for making decisions about the control of AMR in interface areas. Further investigation would also help ensure that the possibility of transmission of AMR genes from wildlife-associated bacteria to human microbiome through livestock or directly is minimized. Whole-genome sequencing could be useful in such investigation, and for resource-limited settings, the availability of online resources for bacterial typing offers rapid classification and source tracing, which is increasingly important in a globalized community. Such online resources offer rapid typing and phylogenetic relatedness linked to antibiotic resistance genes.

## Figures and Tables

**Table 1:** Microbiological, biochemical, and molecular identification of isolates. *Escherichia coli* and closely related isolates from selected animal species as identified using an API 20E Gram-negative Microbial Identification Kit, and molecular identification methods (PCR and Sequencing) using 16E1/16E2/16E3 and P3mod/P5 Primers.

Animal Species	Number of Samples Processed	Total Isolates (% Yield)	Identified Bacteria by Species								
			Biochemical Identification Using API^®^ 20E (*n* = 66)				Molecular Detection Using 16E1/16E2/16E3 and P3mod/P5 Primers (*n* = 65)				
			*Escherichia coli* Group 1 *n* (%)	*Burkholderia cepacian* (%)	*Kluyvera* spp. *n* (%)	Unidentified *n* (%)	*Escherichia coli* spp. *n* (%)	*Escherichia fergusoniin* (%)	*Shigella* spp. *n* (%)	*Enterobacteriaceae bacteriumn* (%)	
Impala	16	15 (93.8)	6 (40)	0	1 (6.7)	8 (53.3)	11 (78.6)	0	3 (21.4)	0	
Buffalo	13	8 (61.5)	5 (62.5)	0	0	3 (37.5)	6 (75)	1 (12.5)	1 (12.5)	0	
Ostrich	7	6 (85.7)	5 (83.3)	0	0	1 (16.7)	5 (83.3)	0	1 (16.7)	0	
Cattle	20	16 (80)	11 (68.8)	0	0	5 (31.3)	13 (81.2)	0	3 (18.8)	0	
Pig	18	15 (83.3)	11 (73.3)	0	0	4 (26.7)	10 (66.7)	0	4 (26.7)	1 (6.7)	
Goat	10	6 (60)	1 (16.7)	1 (16.7)	0	4 (66.7)	3 (50)	0	3 (50)	0	
Total	84	66 (78.6)	39 (59.1)	1 (1.5)	1 (1.5)	25 (37.9)	48 (72.7)	1 (1.5)	15 (22.7)	1 (1.5)	
							**Sequence percentage homology** **(%)**	Minimum	90.00	1st Quantile	98.10
								Mean	98.29	Median	99.00
								Maximum	100.00	3rd Quantile	99.30

**Table 2:** Relative frequencies of isolates exhibiting resistance (R) or intermediate susceptibility (I) to the tested antimicrobials. A dash (–) denotes neither resistance nor intermediate sensitivity was detected (*n* = 63).

Antimicrobial Agent	AMR Detected	All Isolates N = 63		Impala N = 15		Buffalo N = 8		Ostrich N = 6		Cattle N = 16		Pig N = 15		Goat N = 6	
		*E. coli* spp. (*n* = 48) *n* (%)	*Shigella* spp. (*n* = 15) *n* (%)	*E. coli* spp. (*n* = 11) *n* (%)	*Shigella* spp. (*n* = 3) *n* (%)	*E. coli* spp. (n = 6) *n* (%)	*Shigella* spp. (*n* = 1) *n* (%)	*E. coli* spp. (*n* = 5) *n* (%)	*Shigella* spp. (*n* = 1) *n* (%)	*E. coli* spp. (*n* = 13) *n* (%)	*Shigella* spp. (*n* = 3) *n* (%)	*E. coli* spp. (*n* = 10) *n* (%)	*Shigella* spp. (*n* = 4) *n* (%)	*E. coli* spp. (*n* = 3) *n* (%)	*Shigella* spp. (*n* = 3) *n* (%)
Ampicillin	R	17 (27)	3 (4.8)	6 (42.9)	-	-	-	3 (50)	1 (16.7)	7 (43.8)	-	1 (6.7)	1 (6.7)	-	1 (16.7)
	I	10 (15.9)	3 (4.8)	2 (14.3)	-	-	1 (12.5)	-	-	2 (12.5)	1 (6.2)	5 (33.3)	-	1 (16.7)	1 (16.7)
Amoxicillin + Clavulanic Acid	R	5 (7.9)	-	4 (28.6)	-	-	-	1 (16.7)	-	-	-	-	-	-	-
	I	2 (3.2)	-	1 (7.1)	-	-	-	1 (16.7)	-	-	-	-	-	-	-
Ceftazidime	R	9 (14.3)	1 (1.6)	2 (14.3)	-	-	-	1 (16.7)	-	5 (31.2)	-	-	1 (6.7)	1 (16.7)	-
	I	4 (6.3)	-	-	-	-	-	-	-	1(6.2)	-	2 (13.3)	-	1 (16.7)	-
Cefotaxime	R	6 (9.5)	2 (3.2)	2 (14.3)	-	-	-	1(16.7)	1(16.7)	3 (18.8)	-	-	1 (6.7)	-	-
	I	9 (14.3)	1 (1.6)	5 (35.7)	-	-	-	-	-	2 (12.5)	-	2(13.3)	-	-	1 (16.7)
Ceftriaxone	R	4 (6.3)	-	2 (14.3)	-	-	-	1 (16.7)	-	1 (6.2)	-	-	-	-	-
	I	7 (11.1)	2 (3.2)	-	-	1 (12.5)	-	-	1 (16.7)	4 (25)	2 (12.5)	1 (6.7)	-	-	-
Kanamycin	R	6 (9.5)	3 (4.8)	1 (7.1)	-	-	-	2 (33.3)	1 (16.7)	-	-	1 (6.7)	1 (6.7)	2 (33.3)	1 (16.7)
	I	20 (31.7)	5 (7.9)	5 (35.7)	1 (7.1)	3 (37.5)	1 (12.5)	-	-	6 (37.5)	1 (6.2)	4 (26.7)	2 (13.3)	-	-
Amikacin	R	-	-	-	-	-	-	-	-	-	-	-	-	-	-
	I	-	-	-	-	-	-	-	-	-	-	-	-	-	-
Gentamicin	R	-	-	-	-	-	-	-	-	-	-	-	-	-	-
	I	1 (1.6)	-	-	-	-	-	-	-	1 (6.2)	-	-	-	-	-
Imipenem	R	1 (1.6)	-	1 (7.1)	-	-	-	-	-	-	-	-	-	-	-
	I	2 (3.2)	-	1 (7.1)	-	-	-	-	-	2 (12.5)	-	-	-	-	-
Nalidixic Acid	R	3 (4.8)	1(4)	1 (7.1)	-	-	-	-	-	2 (12.5)	-	1 (6.7)	-	-	-
	I	1 (1.6)	-	1 (7.1)	-	-	-	-	-	-	-	-	-	-	-
Ciprofloxacin	R	-	-	-	-	-	-	-	-	-	-	-	-	-	-
	I	1 (1.6)	-	-	-	-	-	-	-	1 (6.2)	-	-	-	-	-
Trimethoprim + Sulfamethoxazole	R	-	-	-	-	-	-	-	-	-	-	-	-	-	-
	I	-	-	-	-	-	-	-	-	-	-	-	-	-	-
Chloramphenicol	R	2 (3.2)	1 (1.6)	-	-	-	-	-	-	-	1 (6.2)	1 (6.7)	-	1 (16.7)	-
	I	-	-	-	-	-	-	-	-	-	-	-	-	-	-

**Table 3:** Distribution of relative frequencies of isolates exhibiting resistance to various antimicrobials. The categories of antimicrobials included in this study are penicillins, cephalosporins, aminoglycosides, quinolones, phenicols, folate pathway inhibitors/sulfonamides, and carbapenems. An isolate was considered multidrug-resistant if it showed resistance to at least one antimicrobial agent each among three or more antimicrobial categories (n = 63).

Number of Antimicrobial Classes	All Isolates		Impala		Buffalo		Ostrich		Cattle		Pig		Goat	
	*E. coli* spp. (*n* = 48) *n* (%)	*Shigella* spp. (*n* = 15) *n* (%)	*E. coli* spp. (*n* = 11) *n* (%)	*Shigella* spp. (*n* = 3) *n* (%)	*E. coli* spp. (*n* = 6) *n* (%)	*Shigella* spp. (*n* = 1) *n* (%)	*E. coli* spp. (*n* = 5) *n* (%)	*Shigella* spp. (*n* = 1) *n* (%)	*E. coli* spp. (*n* = 13) *n* (%)	*Shigella* spp. (*n* = 3) *n* (%)	*E. coli* spp. (*n* = 10) *n* (%)	*Shigella* spp. (*n* = 4) *n* (%)	*E. coli* spp. (*n* = 3) *n* (%)	*Shigella* spp. (*n* = 3) *n* (%)
1	4 (8.4)	-	2 (18.2)	-	-	-	-	-	-	-	2 (20)	-	-	-
2	10 (20.9)	1 (6.7)	1 (9.1)	-	-	-	2 (40)	-	6 (46.2)	-	1 (10)	-	-	1 (33.3)
3	8 (16.7)	1 (6.7)	3 (27.3)	-	-	-	1 (20)	1 (100)	2 (15.4)	-	1 (10)	-	1 (33.3)	-
4	1 (2.1)	1 (6.7)	-	-	-	-	-	-	-	-	-	1 (25)	1 (33.3)	-
5	-	-	-	-	-	-	-	-	-	-	-	-	-	-
6	-	-	-	-	-	-	-	-	-	-	-	-	-	-
7	-	-	-	-	-	-	-	-	-	-	-	-	-	-

## Data Availability

The data presented in this study are available on request from the corresponding author. Sequence data will be available on the DDJB website as soon as the applicable embargo period lapses.

## Supplementary Materials

The following are available online at https://www.mdpi.com/2079-6382/10/3/238/s1, Supplement A: list of detected strains of bacteria isolates.
