# Supplementary material for: Identification of Escherichia coli and Related Enterobacteriaceae and Examination of Their Phenotypic Antimicrobial Resistance Patterns: A Pilot Study at A Wildlife–Livestock Interface in Lusaka, Zambia
Source: Antibiotics (Basel). 2021 Feb 26;10(3):238. doi: 10.3390/antibiotics10030238 (PMC7996741; doi:10.3390/antibiotics10030238)
Supplement: Supplementary file 1 [file antibiotics-10-00238-s001.pdf]

SUPPLEMENT 1

SN	ID	Bacteria spp. Code (for API)	Sequence percentage homology (%)	Name and strain	E Code
1	**BV1**	1	90.3	*E. coli* SD2	E12
2	**BV2**	1	98.1	*E. coli* LD93-1	E16
3	**BV3**	2	99.4	*Shigella flexinerri* SD5	E2
4	**BV4**	1	97.9	*E. coli* O157 CFSAN 076619	E41
5	**BV5**	1	98.8	*E. coli* CICC 10389	E27
6	**BV6**	1	98.8	*E. coli* 824422	E32
7	**BV7**	1	98.6	*E. coli* UWIWRF 0598	E45
8	**BV8**	1	99.5	*E. coli* RHB04-C05	E6
9	**BV9**	1	98.9	*E. coli* LD93-1	E3
10	**BV10**	1	98.9	*E. coli* 216 C-3878	E29
11	**BV11**	1	99.6	*E. coli* 824422	E17
12	**BV12**	1	98.2	*E. coli* LD93-1	E30
13	**BV13**	2	99.3	*Shigella sonnei* SE6-1	E19
14	**BV14**	1	99.6	*E. coli* CCFM 8341	E33
15	**BV15**	2	99.1	*Shigella sonnei* SE6-1	E13
16	**BV16**	1	98.5	*E. coli* NF55	E51
17	**PG1**	1	99	*E. coli* PB7DCRUSTSK	E24
18	**PG2**	1	98.5	*E. coli* EGE 4946786-102	E50
19	**PG3**	1	98.1	*E. coli* KCJ 5116	E44
20	**PG4**	1	97.6	*E. coli* PB7DCRUSTSK	E36
21	**PG5**	1	98.3	*E. coli* 388808	E23
22	**PG6**	1	99	*E. coli* HUAIAN 1612	E35
23	**PG7**	1	98.9	*E. coli* LD93-1	E7
24	**PG8**	2	99.3	*Shigella sonnei* SE6-1	E39
25	**PG9**	2	98.8	*Shigella sonnei* SE6-1	E40
26	**PG10**	1	99.2	*E. coli* PB7DCRUSTSK	E56
27	**PG11**	2	99.1	*Shigella* spp. NCCP-460	E42
28	**PG12**	1	99.2	*E. coli* FORC-081	E37
29	**PG13**	1	97.6	*E. coli*	E21
30	**PG14**	3	99.5	*Enterobacteriaceae bacterium* 28W431	E14
31	**PG15**	2	100	*Shigella sonnei* SE6-1	E11
32	**OS1**	1	99.3	*E.* coli RHB04-C05	E10
33	**OS2**	1	99.8	*E. coli* YJ4	E5
34	**OS3**	1	99.6	*E. coli* PGB 01	E26
35	**OS4**	2	99.1	*Shigella sonnei* SE6-1	E43
36	**OS6**	1	96.7	*E. coli* PNR 347	E25
37	**OS7**	1	99.2	*E. coli* LD93-1	E8
38	**G1**	1	90	*E. coli* DPR 12	E48
39	**G3**	2	99.5	*Shigella sonnei* SE6-1	E22
40	**G4**	2	98.6	*Shigella sonnei* SE6-1	E46
41	**G7**	2	99.3	*Shigella sonnei* SE6-1	E62

SN	ID	Bacteria spp. Code (for API)	Sequence percentage homology (%)	Name and strain	E Code
42	**G9**	1	91.2	*E. coli* UVF-153	E15
43	**G10**	1	98.8	*E. coli* WP5-W18-ESBL11	E61
44	**B1**	2	99.2	*Shigella sonnei* SE6-1	E66
45	**B2**	1	99.4	*E. coli* DPR 12	E58
46	**B3**	1	99.2	*E. coli* FR 153	E18
47	**B4**	0	95.6	*E. fergusonii* 2438	E20
48	**B5**	1	99.7	*E. coli* F3-1-9	E52
49	**B8**	1	98.8	*E. coli* CCFM 8341	E47
50	**B12**	1	99.6	*E. coli* G1/2	E59
51	**B13**	1	94.9	*E. coli* LD 26-1	E28
52	**I1**	2	97.8	*Shigella sonnei* SE6-1	E34
53	**I2**	1	95	*E. coli* RM 7190	E9
54	**I3**	1	99	*E. coli* FR-2	E54
55	**I4**	1	97.3	*E. coli* AR 0149	E38
56	**I5**	1	99.2	*E. coli* LD93-1	E4
57	**I6**	1	99.3	*E. coli* HUAIAN 141	E65
58	**I7**	1	98.1	*E. coli* ECPF-16	E63
59	**I8**	1	98.1	*E. coli* CCFM 8341	E31
60	**I9**	1	99.5	*E. coli*	E1
61	**I11**	1	99.1	*E. coli* WP2-S18-ESBL-07	E57
62	**I12**	2	99.3	*Shigella sonnei* SE6-1	E55
63	**I13**	2	99.3	*Shigella sonnei* SE6-1	E60
64	**I14**	1	96.4	*E. coli*	E53
65	**I15**				E49
66	**I16**	1	98.5	*E. coli* CCFM 8341	E64

KEY

ANIMAL SPECIES ABBREIATIONS

BV CATTLE (*Bos taurus*)
PG PIG (*Sus scrofa*)
OS OSTRICH (*Struthio camelus*)
G GOAT (*Capra hircus*)
B BUFFALO (*Syncerus caffer*)
I IMPALA (*Aepyceros melampus*)

BACTERIA SPECIES CODE

0 *E. fergusonii*
1 *E. coli* group
2 *Shigella* species
3 *Enterobacteriaceae bacterium*
